# Multi-modal imaging using scanning diffraction and microscopy to elucidate tissue architecture

**DOI:** 10.1063/4.0001065

**Published:** 2025-10-27

**Authors:** Rama S. Madhurapantula

**Affiliations:** Illinois Institute of Technology, Chicago, IL, USA

## Abstract

Accurate annotation of structural and functional details within diverse tissues is crucial for scientific advancement. Multi-modal and multi-scale imaging techniques, capable of revealing material and mechanical properties, are increasingly vital for biomedical innovation. Driven by advancements in high-speed imaging hardware and software, multi-modal imaging has emerged as a cornerstone methodology for investigating both healthy and diseased biological tissues at multiple scales.

Scanning Diffraction Imaging (also known as diffraction imaging (DI)) utilizes X-ray diffraction from a microbeam to raster scan molecular packing within tissue samples, revealing the distribution and orientation of ordered constituents (ex: collagen, myelin, and amyloids). Analysis of diffraction patterns yields: 1) integrated intensity proportional to the amount of diffracting material at that tissue location, 2) d-spacing that can reveal the identity of the tissue component 3. the orientation of the long axis of the diffracting material (which direction the molecules are “pointing") 4. the degree of disorientation around this axis, determined by the angular width of the meridional arcs. This presentation outlines the use of DI to determine tissue architecture and physical properties of various animal and human tissues. It will demonstrate its application in multi-modal imaging when combined with traditional microscopy and material testing. It will also outline the development and use of Musclex – DI – a software program used to analyze and visualize DI data. This integrated approach elucidates long-range structural changes and their correlation to functional deficits, offering a powerful tool for developing high-resolution, multi-scale tissue models and understanding structure-function relationships in biological tissues.

FIX1

